# Non-Coding microRNAs as Novel Potential Tumor Markers in Testicular Cancer

**DOI:** 10.3390/cancers12030749

**Published:** 2020-03-22

**Authors:** Manuel Regouc, Gazanfer Belge, Anja Lorch, Klaus-Peter Dieckmann, Martin Pichler

**Affiliations:** 1 Research Unit of Non-Coding RNAs and Genome Editing in Cancers, Medical University of Graz, 8010 Graz, Austria; manuel.regouc@stud.medunigraz.at; 2 Division of Clinical Oncology, Department of Medicine, Comprehensive Cancer Center Graz, Medical University of Graz, 8010 Graz, Austria; 3 Faculty of Biology and Chemistry, University of Bremen, 28359 Bremen, Germany; belge@uni-bremen.de; 4 Department of Medical Oncology and Haematology, University Hospital Zurich, 8091 Zuerich, Switzerland; anja.lorch@usz.ch; 5 Department of Urology, Asklepios Klinik Altona, 22763 Hamburg, Germany; dieckmannkp@t-online.de; 6 Department of experimental therapeutics, The University of Texas MD Anderson Cancer Center, Houston, TX 77030, USA

**Keywords:** testicular cancer, tumor markers, microRNAs

## Abstract

Testicular cancer is an important disease with increasing incidence and a high burden of morbidity and mortality in young men worldwide. Histological examination of the testicular tissue after orchiectomy plays an important role alongside patient history, imaging, clinical presentation and laboratory parameters. Surgical procedures and chemotherapeutic treatment provide a high chance of cure in early stages, though some patients in advanced stages belonging to a poor risk group experience cancer-related death. Though conventional serum-based tumor markers, including α-fetoprotein (AFP), the β-subunit of human chorionic gonadotropin (β-hCG), and lactate dehydrogenase (LDH), are useful as prognostic and diagnostic biomarkers, unfortunately, these tumor markers only have a sensitivity of about 60%, and in pure seminoma even lower with about 20%. Therefore, the development of new tumor markers is an important and intensively ongoing issue. The analysis of epigenetic modification and non-coding RNA microRNAs (miRNAs) are carrying most promising potential as tumor markers in future. miRNAs are small RNAs secreted by testicular tumor cells and circulate and be measurable in body fluids. In recent years, miRNAs of the miR-371-373 cluster in particular have been identified as potentially superior tumor markers in testicular cancer patients. Studies showed that miR-371a-3p and miR-302/367 expression significantly differ between testicular tumors and healthy testicular tissue. Several studies including high prospective multi-center trials clearly demonstrated that these miRNAs significantly exceed the sensitivity and specificity of conventional tumor markers and may help to facilitate the diagnosis, follow-up, and early detection of recurrences in testicular cancer patients. In addition, other miRNAs such as miR-223-3p, miR-449, miR-383, miR-514a-3p, miR-199a-3p, and miR-214 will be discussed in this review. However, further studies are needed to identify the value of these novel markers in additional clinical scenarios, including the monitoring in active surveillance or after adjuvant chemotherapy, but also to show the limitations of these tumor markers. The aim of this review is to give an overview on the current knowledge regarding the relevance of non-coding miRNAs as biomarkers in testicular cancer.

## 1. Introduction

Testicular cancer is one of the most important neoplasms in adolescent and young adults with the highest incidence between the ages of 15 to 35 years [1]. The histological distinction of different testicular tumors is crucial for further therapies and prognosis. The International Agency for Research in Cancer of the World Health Organization (WHO) classified these tumors and has divided them into numerous sub-groups. In addition to sex cord-stromal tumors (Leydig cell tumor and Sertoli cell tumor), testicular germ cell tumors (TGCTs) play the most important clinical role due to frequent occurrence and the potential to cure malignant tumors even in the metastatic setting since the early introduction of Cisplatin-containing chemotherapy [2,3]. Mostly unknown environmental factors and some well-known risk factors like undescended testes and a positive family history led to a doubling of the incidence over the last forty years [3]. Despite the high cure rates achieved with the introduction of cisplatin-containing regimen, many short-term as well as long term toxicities are described. The proper management include the risk-stratification with regard to thromboembolism, cardiovascular toxicity and neutropenic complications [4,5,6]. Even elderly patients with metastatic TGCT can achieve high cure rates similar to younger patients if they tolerate risk-adapted chemotherapy [7].

While clinical examination, scrotal ultrasound and high-resolution computed tomography (CT) are the diagnostic mainstays, the clinical relevance of serum tumor markers is unsurpassed. α-fetoprotein (AFP) and the β-subunit of human chorionic gonadotropin (β-hCG) and Lactate dehydrogenase (LDH) are measured in a standardized way for any suspect testicular cancer [1]. The high sensitivity and specificity of β-hCG and AFP allow to make statements about the diagnosis, histology, classification, and prognosis of TGCTs. These markers are also useful to monitor patients and to track the course of the disease after active surveillance or disease recurrence. However, depending on the tumor type and co-morbidities, false positive or false negative results can occur [8,9]. Therefore, a lot of effort has been spent to discover and develop novel tumor markers with improved specificity and sensitivity [10]. One promising approach is to analyze blood for circulating microRNAs (miRNAs). miRNAs are single stranded molecules with a length of 19-22 nucleotides with a specific sequence that may have significant effects on carcinogenesis as post-transcriptional regulators. Especially miRNAs of the miR-371-373 cluster (but also others, including miR-223-3p, miR-449, miR-383, miR-514a-3p, miR-199a-3p, miR-214) are considered potential new tumor markers [11]. Therefore, the search and validation of these novel tumor markers in different disease settings is currently intensively followed worldwide [12]. In this review, the clinical relevance of already available serum markers is discussed and compared with new markers and detection methods, such as the quantification of miRNAs, in order to highlight new diagnostic options.

## 2. Discussion

### 2.1. Biomarkers as Novel Diagnostic Tools

The serum tumor markers β-hCG, LDH and AFP are already part of the TNM classification (UICC, 2016, 8th edition) thus influence the staging of tumors in clinical practice [13]. The problem with these markers is the lack of sensitivity and specificity: only 20–30% of pure seminomas produce β-hCG and only every second non-seminoma TGCT has elevated LDH, AFP, or β-hCG levels [9]. In addition, false-elevated levels may be caused by other solid tumors (pancreas, liver, stomach, kidneys) or by smoking marijuana, which further complicates follow-up [14]. 

Due to the uncertainty of current biomarkers, additional markers specifically produced by TGCTs are sought to minimize the number of false negative/positive results. This includes the study of epigenetic alteration of the tumors. Altered DNA methylation and histone modifications, changes in the chromatin remodeling system, and specifically miRNAs, may provide predictive and prognostic information about TGCTs in the future [15]. Several studies have already shown that the cluster miR-371-373 and miR-367 have a significantly higher sensitivity and specificity than the conventional serum markers [16].

In the next part, conventional markers are compared with the miRNA clusters to make statements about the new, possibly better, tumor markers.

### 2.2. Alpha-Fetoprotein

Alpha Fetoprotein (AFP) is a 609 amino acid (aa) long glycoprotein that consists of a signal peptide and a main chain. It belongs to the group of serum albumins and occurs mainly as a monomer. Copper, nickel, fatty acids and, to a lesser extent, estrogens can bind to AFP [17]. AFP has a serum half-life of 5–7 days and normalizes after 3–4 weeks in TGCT patients after therapy [18]. AFP reaches its maximum concentration at week 13 during pregnancy and is mostly produced by yolk sac [19]. For this reason, elevated AFP levels are not found in pure seminoma or trophoblastic tumors, but in 90% of yolk sac tumors and sometimes in embryonal carcinomas. Nonetheless, liver disease, other solid tumors with AFP production, or a genetic persistence of AFP may also lead to increased levels [14].

Results by Klepp et al. have shown that normal AFP levels preoperatively indicate a lack of yolk sac histology [20]. AFP often cannot predict a recurrence in Stage I TGCTs, but allows a prognosis estimation and monitoring together with LDH and β-hCG which is useful in advanced tumors. AFP is the most important conventional marker in late relapses of non-seminomatous tumors [14]. 

### 2.3. β-subunit of Human Chorionic Gonadotropin

Human chorionic gonadotropin (HCG) is a peptide hormone that consists of a nonspecific alpha subunit, which is also part of the thyroid stimulating hormone (TSH), luteinizing hormone (LH) and follicle stimulating hormone (FSH), and a specific 137 aa beta subunit, of which several protein variants exist [21,22]. The serum half-life of β-hCG is approximately 18–36 hours [18].

The syncytiotrophoblasts of the placenta produce β-hCG in pregnant women. These cells can also occur in testicular cancer and produce β-hCG, so that pathologically high serum levels can be measured. Increased levels are always found in choriocarcinomas, 40–60% in embryonic carcinomas, and 10–30% in seminomas. β-HCG can be used for prognosis estimation and further monitoring. If the β-hCG level increases despite therapy, a complete staging including the contralateral testis and brain should be examined for possible metastasis or neoplasm. Elevated levels over 6 weeks are associated with poor survival [14,21]. 

An orchiectomy can lead to hypogonadism with a reduced testosterone level, which causes increased levels of LH by feedback with the pituitary gland. Cross-reactions of LH in β-hCG assays can simulate elevated levels. During the first days of treatment of TGCTs with chemotherapy may transiently increase the serum marker due to the destruction of the tumor cells (“surge phenomenon”). Like AFP, β-hCG can be produced by other tumors and thus influence diagnostics and should be considered when decision making is based on this tumor marker [23].

### 2.4. Lactate Dehydrogenase

L-lactate dehydrogenase (LDH) is an approximately 35,000 kDa enzyme that is ubiquitous in the cytoplasm of cells. LDH catalyzes the reversible reaction of pyruvate to lactate with the help of the cofactor NAD^+^/NADH and is involved in energetic processes such as glycolysis and gluconeogenesis [24,25]. The half-life of LDH is approximately 24 h [26].

In general, cell death and the consequent leakage of LDH from the cell may increase serum levels. LDH is therefore very unspecific, since cell lysis can also occur in myocardial infarction, liver and kidney diseases, hemolysis, as well as in other cancers [23]. Elevated LDH levels are associated with the tumor lysis syndrome that can occur due to rapid disintegration of tumor cells after treatment with chemotherapeutics. LDH is rather nonspecific and may only have an impact on the treatment of TGCTs in conjunction with other markers and diagnostic criteria [27].

### 2.5. Placental Alkaline Phosphatase

Placental alkaline phosphatase (PLAP) is one of four isoenzymes (intestinal, germ cell, non-specific type) that is not used in TNM classification due to the high number of false positive results [28,29]. Physiologically, PLAP is produced by primordial germ cells (PGCs), syncytiotrophoblastic cells and gonocytes. Also, in seminomas, yolk-sac tumors and embryonal carcinomas, expression can take place whereas in teratomas elevated PLAP values are usually not found. Due to the low sensitivity, PLAP is no longer recommended by guidelines, though in seminoma, the sensitivity is higher than the conventional used markers. Nevertheless, the unsolved issue with PLAP is mainly due to the increased PLAP secretion of alveolar tissue in smokers, which allows 10-fold increased levels to be measured, and is a common source for misleading results [14,28].

### 2.6. DNA Methylation and Histone Modification

The previously described conventional TGCT markers, namely AFP, β-hCG, LDH, and PLAP, can be determined by serological examination. Another possibility is direct examination of tumor cells using immunohistochemical diagnostics. Testicular tumors have altered epigenetic patterns compared to somatic cells, which could subsequently be considered as tissue-based or circulating tumor markers [15]. 

In the development of the testis, hypomethylated germ cells appear in the early phase and hypermethylation in mature phases. Genomic hypomethylation is generally found in tumors and hypermethylation at certain loci. The degree of methylation is highly dependent on the differentiation of TGCTs. Hypomethylation increases with the dedifferentiation of testicular cancer. There are also differences between the tumor entities: Embryonic carcinomas are moderately methylated, while teratomas and choriocarcinomas have highly methylated genomes [30]. The degree of methylation of CpG islands differs in that most of the chromatin in TGCTs lose their strong methylation, while other normally not methylated CpG sites are hypermethylated [31]. The CpG islands of *XIST* (X-inactive specific transcript) gene (encoded for a long non-coding RNA) are methylated in somatic cells and unmethylated in TGCTs. Non-coding regions such as Long Interspersed Nuclear Elements (LINE1) and also promoters may have different methylation patterns in testicular cancer [15,32]. Altered CpG methylation was detected by Ellinger et al. in cell-free circulating tumor DNA in serum. Hypermethylation of *APC*, *p16,* and *PTSG2* genes show high specificity and may be potential biomarkers [33]. Moreover, 5-methylcytosine of the CpG islands can also be detected by immunohistochemistry [34].

Methylation and other modifications also occur in histones, which reorganize the chromatin. These are necessary to maintain cell integrity and prevent transformation to TGCTs. The changes in chromatin are manifold and make it difficult to identify tumor markers. Overexpression of DNA methyltransferase I (DNMT1) may result in silencing of tumor suppressor genes [35]. Altered histone methylations increase the expression of proto-oncogene *POU5F1*, which is detectable in embryonal carcinomas and seminomas [36]. Silencing of tumor suppressor *RASSF1A* was observed by histone modifications (loss of trimethylation of H3K9) at the promoter. Nonetheless, the current data is insufficient to use histone modification as a biomarker [15].

### 2.7. MicroRNAs as Novel Emerging Biomarkers

MicroRNAs (miRNAs) are a group of molecules that are currently under intense research and have high likelihood for potential tumor markers [12]. In contrast to the long non-coding RNAs, miRNAs belong to the group of short non-coding RNAs and have been involved in the pathogenesis of many types of cancer [37,38,39]. This group of RNAs has an average length of 22 nucleotides that do not code for proteins (non-coding RNA). miRNAs attach mRNAs by complementary base binding and can post-translationally control the expression of proteins in cells [12,40]. miRNAs affect a variety of molecular processes and are necessary for the proper development of tissues, drug resistance and immune response [41,42,43,44]. miRNAs can prevent the transformation to cancer, but in the case of abnormal expression patterns, they may also promote it. Thus, miRNA clusters were found that are only expressed higher in TGCTs regardless of histologic type, age of patients and localization [27]. miRNAs are extracellular signal molecules for cell-cell communication, which are also secreted by tumor cells in the blood and are thus easily detectable. miRNAs are resistant to RNase A and are therefore stable in serum [45]. The detection of miRNA was also successful in other body fluids such as urine, saliva, breast milk, seminal plasma, and cerebrospinal fluid. miRNAs are generally present in two states, either in combination with the AGO2 protein, or are found unbound in vesicles [46,47,48]. They are secreted by cells in the context of regulatory processes or intracellular communication, but can also be released by cell death. miRNAs can affect other cells in an autocrine, paracrine or endocrine manner. The penetration of miRNAs into the cell is not yet fully understood. Presumably, this takes place via endocytosis or a receptor-dependent uptake [41]. There have been several examples of miRNAs and their respective functions, but just to highlight some of them, Schwarzenbacher and colleagues identified miR-1287-5p as significantly down-regulated in breast cancer and cancer stem cells. They were able to show that miR-1287-5p significantly decreased cellular growth, cells in S phase of cell cycle, anchorage-independent growth, and tumor formation in vivo. By identifying PI3Kinase pathway as a main regulated pathway, they also suggest a possible pharmacological link to miR-1287-5p [49]. Another example of the involvement of miRNAs in human cancer comes from a work published by Pichler and colleagues. In their genome-wide analysis of the Cancer Genome data set, the authors identified six miRNAs (miR-92b-3p, miR-188-3p, miR-221-5p, miR-331-3p, miR-425-3p, and miR-497-5p) as strong predictors of survival in colorectal cancer. One of them, miR-188-3p increased the migratory behavior of colorectal cancer cells in vitro and metastases formation in vivo. Furthermore, the authors could link miR-188-3p to MLLT4, a novel identified player involved in colorectal cancer cell [50]. 

miR-302, miR-367 and miR-371-373 clusters already provide high specificity (>99%) and are the most promising candidates for TGCT markers [11,27,34].

#### 2.7.1. miR-302 Cluster

miR-302 cluster plays an important role in the regulation of the cell cycle in embryonic (ESC) and pluripotent stem cells (PSC). miR-302 can interact with cell cycle promoters and inhibitors, thereby affecting the rapid proliferation of these cells [51]. In addition, they regulate epigenetic processes (histone methylations) and signaling cascades such as the Akt/PKB signaling pathway [52]. High miR-302 expression is mainly found in teratomas where it suppresses the oncogenic driver Akt. As a consequence, there is less inhibition of the pluripotent transcription factor OCT4 by Akt. Conversely, downregulation of miR-302 or overexpression of Akt in PSCs results in low expression of OCT4. High OCT4 levels are necessary for the pluripotent capabilities of PSCs, but are also a factor that promotes teratoma formation. miR-302 also inhibits the expression of other cell cycle inhibitors (CDK2, CDK4) and thus accelerates the transition from G1 phase to S phase. Expression in ESCs decreases rapidly after differentiation [51,53,54].

A study published by Das et al. has shown that miR-302 cluster is highly expressed specifically in embryonic carcinomas. In contrast to low expression of miR-302 in colon, stomach and liver carcinomas, only in TGCT patients have been found increased miR-302 in the blood [55,56,57]. In TGCT cell lines treated with cisplatin, expression of miR-302a-3p, miR-302b-3p and miR-302c-3p decreased [53]. Sprouty RTK Signaling Antagonist 4 (SPRY4] is a potential oncogene that may be overexpressed in TGCT and affect the PI3K/Akt signaling pathway. Inhibition of miR-302 suppresses SPRY4, which subsequently decreases cell growth and invasion [53]. These results indicate a possible role of miR-302 in the development of TGCTs [58].

The miR-302 cluster consists of four individual miRNAs (miR-302a, miR-302b, miR-302c, miR-302d), which differ in part only by a few nucleotides. Therefore, precise methods are needed to differentiate these miRNAs from one another during serum analysis. Murray et al. found elevated miR-302 levels in blood tests in all TGCT patients studied. When using a multiplex real-time polymerase chain reaction (qRT-PCR), no cross-reactions between the miRNAs were detected [59]. However, more studies are needed to confirm the potential of miR-302 as a tumor marker.

#### 2.7.2. miR-367

miR-367 is a miRNA that is closely associated with the miR-302 cluster and therefore often summarized (miR-302/367 cluster). miR-367 differs slightly in sequence with the miR-302 cluster, but they have similar mRNAs as targets [52]. The common seed sequence of miR-302a-d is “AAGUGCU”, while miR-367 shares the sequence “AUUGCAC” with miR-92a-1 and miR-92a-2. The physiological functions of miR-302/367 cluster include the control of nodal signaling pathway and the cell cycle. Decreased miR-302/367 expression was found in GCTs and yolk sac tumors and correlates negatively with the expression level of tumor suppressor protein P63 [60].

In a study by Syring et al., the miR-367-3p serum level was determined in 76 patients with TGCT and in 84 healthy patients. TGCT patients had a significantly increased miR-367-3p level. However, there was no difference from miR-302 level between both groups. Stage I and II TGCTs had significantly lower miR-367-3p levels than advanced tumors (Stage III). miR-367-3p was more elevated in seminomas than in non-seminomas. After performing orchiectomy, miR-367-3p decreased or was no longer detectable [61]. Another study in pediatric germ cell tumors analyzing cerebrospinal fluid and serum demonstrated that a four-serum miRNA panel (miR-371a-3p, miR-372-3p, miR-373-3p and miR-367-3p) possesses high sensitivity/specificity for diagnosing pediatric extracranial malignant GCT, allows early detection of relapse of a testicular mixed malignant germ cell tumor, and distinguishes intracranial malignant germ cell tumors from intracranial non-germ cell tumours at diagnosis [62]. Another study reported impressive data by including miR-373-3p and 367-3p serum levels to detect germ cell tumors in 250 patients with an area under the curve (AUC) of 0.962, with a 90% sensitivity and 91% specificity. Their assay, which they called ampTSmiR, was not suitable to detect pure teratoma as well as the precursor of TGCC, i.e., germ cell neoplasia in situ (GCNIS) [63]. There was no detection in blood in patients with benign testicular lesions [61]. However, miR-371-3p seems to outperform the high sensitivity of miR-367-3p in germ cell tumor patients [64]. Though some retrospective studies propose superiority of miR-371-3p to predict viable tumor tissue after chemotherapy and disease [65] recurrence after curative treatment [16], others propose a value for miR-367-3p in indicating chemotherapy-refractory disease [66]. Summarizing, a definitive answer whether miR-367-3p will add additional value when supplemented to miR-371-3p or used under certain clinical scenario has still to be clarified.

#### 2.7.3. miR-371-373 Cluster

The miR-371-373 cluster includes one of the most promising miRNAs that could emerge as a routinely used biomarker in the diagnosis of testicular cancer. This cluster consists of three miRNAs (miR-371, miR-372, and miR-373), all located at chromosome 19. Aberrant (up-regulation) expression of miR-371-373 was found mainly in TGCT patients [67], but this cluster has also a prognostic value in other types of cancers or diseases: miR-371 and miR-372 may play a role in the diagnosis of gastric and pancreatic cancer, while miR-373 influences the metastasis of breast cancer by regulating migration and invasion [68]. MiR-371-373 and miR-302 clusters are universally overexpressed in malignant GCTs and coordinately down-regulate mRNAs involved in biologically significant pathways [69].

The miR-371-373 cluster is linked to the Wnt/β-catenin pathway via a feedback loop and helps maintain the characteristics of stem cells. The substitution of mutant p53 and inhibition of Ras-induced senescence are functions of miR-371-373 up-regulation in TGCTs [70]. Patients with p53-positive TGCTs and upregulated miR-371-373 are less resistant to chemotherapy and radiation treatment [70,71,72,73].

#### 2.7.4. miR-371a-3p

Numerous studies described miR-371a-3p as a suitable tumor marker in TGCT patients in follow-up, recurrence detection, prognosis, diagnostics, and staging. 

First evidence for the potential of this miRNA for TGCT detection came from Dieckmann et al. in a small cohort of TGCT patients. In this study, the authors reported much higher serum levels of miRNA-371-3p in TGCT patients than in healthy controls. Moreover, the serum levels dropped post-operatively in stage I patients, and after systemic chemotherapy treatment in advanced stages, though no correlation was found between the extent of miRNA expression in tissue and the values measured in matching serum [74].

In a study of 28 patients with seminoma or non-seminomatous stage I tumors, significantly increased levels of miR-371 and miR-372 were found, while miR-302 and miR-373 cluster showed no significant differences compared to the control group [75].

A study performed by Vilela-Salgueiro et al., described the differential expression of miR-371a-3p in various testicular tumor entities. Therein, 119 patients with TGCT were examined after orchiectomy and miR-371a-3p was extracted from the histological sections of the tumors and quantified by qRT-PCR. In tumors with germ cell neoplasia in situ (GCNIS) as precursor, as well as in non-GCNIS tumors, no association was found between the expression of miR-371a-3p and the level of serum markers, tumor stage and age of the patients [73]. When comparing with the control group (normal testis tissue), significantly increased miR-371a-3p levels were found in TGCTs. In seminomas, the highest expression was measured, followed by embryonic carcinomas. In general, TGCTs with GCNIS as precursor displayed higher expression than non-GCNIS tumors. The expression level of miR-371a-3p was only slightly increased in teratomas. The prepubertal and post-pubertal types of yolk sac tumors showed no difference in expression. Nonetheless, higher levels of miR-371a-3p expression were found in all tumor types than in healthy tissue [73].

Another study examined serum levels of miR-371a-3p in patients with GCNIS. Therein, 50% of patients with GCNIS had elevated serum levels of miR-371a-3p, while miR-367 was not elevated. After treatment of the patients, miR-371a-3p dropped to normal. Since only one in two patients with precursors of TGCTs had elevated serum levels and there were also contradictory results reported from other research groups, miR-371a-3p cannot replace a biopsy to differentiate between pre-cursor lesions and TGCT, but it may help with unclear histological results [63,76]. A recently published case report suggest a discriminative power of miR-371a-3p to differentiate between unspecific AFP elevations [77].

Spiekermann et al. examined the serum miR-371a-3p levels from the blood of the testicular vein and cubital vein in TGCT patients and compared them to patients with testicular disease (hydrocele, epididymitis) or other solid tumors. Patients with Stage I TGCT had significantly increased miR-371a-3p levels compared to patients with other solid tumors, but there was no substantial difference in GCNIS patients. Interestingly, in the testicular vein blood samples, miR-371-3p levels were at a much higher level than in the cubital vein samples, indicating a possible main origin of the circulating miRNA. The serum values also correlate with the tumor stage, so that the highest miR-371a-3p values were found in serum and pleural effusion of stage III TGCT patients [78,79]. After orchiectomy, miR-371a-3p serum levels decreased by over 97% within 24 hours and over 99% in 72 hours. This is due to the short half-life of miRNAs, which was observed for both, seminomas and non-seminomatous tumors [80]. In advanced TGCT similar results were obtained, however, the influence of metastases on the miR-371a-3p level was not fully examined [80]. 

The expression of miR-371a-3p in the seminal plasma was not significantly different between TGCT patients and healthy patients. However, the miR-371a-3p level in the seminal plasma compared to the serum in healthy men is greatly increased and correlates directly with the sperm concentration. Thus, miR-371a-3p not only plays a role in TGCT diagnostics, but could also serve as a biomarker for male infertility [81].

A prospective study investigated serum levels of miR-371a-3p, miR-372, and miR-373-3p in 24 patients with seminoma and non-seminomatous tumors. All three miRNAs had significantly higher serum levels before orchiectomy than postoperatively, with miR-371a-3p decreasing most rapidly after surgery. In two cases there was no decrease of miR-372 and miR-373-3p. As already described by Spiekermann et al., elevated serum levels in the blood from the testicular vein were also found here. miR-371-373 expression decreased in patients with Stage II/III TGCT after chemotherapy [74]. 

In a large-scale prospective multicenter study, the serum levels of miR-371a-3p in a total of 616 patients with TGCT at different stages and 258 healthy men were recorded and compared with AFP, β-hCG, and LDH. As with the previously described studies and validated for the first time in a prospective manner, increased miR-371-3p levels were found in all tumor subtypes except teratoma. miR-371-3p levels were highest in seminoma and increase with a higher stage of the tumor disease. At each stage of the tumor there was a decrease in serum miR-371a-3p after chemotherapy or orchiectomy, but the miR-371-3p level decreased more rapidly in earlier stages. This can be attributed to metastases, which probably express miR-371a-3p. In two patients who did not survive due to tumor progression, increasing miR-371a-3p levels were measured, so the serum level is likely to be associated with tumor size and disease progression [82].

These results confirmed the importance of miR-371a-3p in the diagnosis of metastases and monitoring of TGCTs treatment course. However, there are also limitations of this marker highlighted in this study: In stage I teratomas, miR-371a-3p could not be detected. In pure seminomas less than 1 cm in size, expression was found in only about 60% [82].

The majority of all TGCT patients are cured by the treatment, but in about 10% of cases there is a recurrence [3]. The monitoring of miR-371a-3p serum levels may be useful in the early detection of recurrences. In a retrospective study, seminoma patients with recurrence were found to have elevated miR-371a-3p serum levels compared to patients without relapse. This significant difference was not found for miR-367-3p, AFP, β-hCG and LDH. In teratoma patients, miR-371a-3p was not detected. Measurement of miR-371a-3p at defined pre-treatment times, after orchiectomy and chemotherapy, as well as in follow-up, may allow early detection of recurrences, especially in seminoma patients [16].

Mego et al. described the influence of miR-371a-3p on progression-free-survival (PFS) and overall survival (OS) of TGCT patients. In general, an association was again found between pre-therapeutic miR-371-3p levels and retroperitoneal lymph node metastases, distant metastases, and high stage. In non-seminomatous tumors, a negative miR-371a-3p level before treatment was associated with better OS and PFS. Similarly, negative miRNA levels in advanced tumors show a positive influence on survival. No association was found between miR-371a-3p and conventional tumor markers. However, the prognostic value of negative tumor markers in seminoma patients was questionable in this study [83]. 

Discussing the quantification of miR-371-3p, mainly RT-qPCR-based assays were used. The samples are usually normalized by deltaCt method via a reference gene, ideally which is an endogenous miRNA. miR-20a-5p, miR-30b-5p, miR-93-5p and miR-191-5p were proposed as reference miRNAs and were commonly used in the analysis of miR-371a-3p and miR-372-3p [62,64,84]. Hemolysis of red blood cells during sampling can lead to misleading results in the measurement. Measurements showed that hemolysis increased the concentration of reference miRNAs in the serum. As a further consequence, the determination of miR-371a-3p and miR-372-3p (which themselves are not affected by hemolysis) can be falsified, thus adversely affecting the diagnosis. Therefore, hemolysis and any kind of contamination of the samples should be avoided, or other miRNAs should be considered as endogenous calibrators and normalizers [85].

In summary, miR-371a-3p is currently considered a very suitable tumor marker for TGCTs, although further prospective studies have to follow in order to identify advantages and limitations [86].

#### 2.7.5. miR-372/373

In contrast to miR-371a-3p, miR-372 and miR-373 seem to be inferior biomarkers with a minor role. Although the expression of miR-372 and miR-373 differ in patients with healthy testes and those with TGCT, the diagnostic performance of miR-371a-3p is much better. In a study of different serum miRNAs in TGCTs, the highest measurable levels were miR-371a-3p and these correlated most with the occurrence of testicular tumors (miR-371a-3p > miR-372a-3p > miR-373a-3p) [62].

Although miR-372 and miR-373 are unlikely to play a role as future tumor markers, they indeed play important role in tumorigenesis. Up-regulation of miR-372/373 leads to the suppression of Large Tumor Suppressor Kinase 2 (LATS2) and subsequently to indirect inhibition of p53, allowing mutations to accumulate in the tumor cell [68,70].

#### 2.7.6. miR-517/519

miR-517a-3p, miR-519a-3p, and miR-519c-3p are three miRNAs that are part of the chromosome 19 miRNA cluster (C19MC), which is located near the miR-371-373 cluster [87]. Overexpression of these miRNAs has already been found in various tumors and is associated with increased invasion, migration and poor overall survival [88,89]. Flor et al. compared the expression of miR-517a-3p, miR-519a-3p, and miR-519c-3p in TGCTs with healthy testicular tissue. The expression in stage I seminoma and mixed tumors with a teratoma component was lower or the same as in normal testes. High expression was found in advanced tumors (stage III mixed tumors) and non-seminomatous tumors. The level of miR-517a-3p in the serum decreased after the tumor was removed. The data show that the expression of miR-517a-3p, miR-519a-3p and miR-519c-3p depends on the histological type and the clinical stage [90]. The role as a potential biomarker for non-seminomatous and advanced tumors would have to be examined in further studies.

#### 2.7.7. miR-223-3p

miR-223-3p is an important miRNA that plays a role in cell growth and apoptosis of tumor cells [91,92]. Altered expression patterns of miR-223-3p have already been observed in esophageal tumors, gastric tumors and in acute T-cell lymphoblastic leukemia [93,94,95]. In TGCTs, increased expression was observed in comparison with healthy testicular tissue. F-box/WD repeat-containing protein 7 (FBXW7) is a tumor suppressor and one of the major targets of miR-223-3p. FBWX7 is part of a protein complex that promotes the degradation of oncoproteins. Increased expression of miR-223-3p correlates with a decrease in FBWX7, which subsequently influences the progression of TGCTs. In TGCTs, overexpression of miR-223-3p leads to the inhibition of apoptosis via FBWX7 [91,94]. The influence of miR-223-3p in gastric carcinoma on the sensitivity of chemotherapeutic agents may also be transferred to TGCTs. There are currently no studies on the use of miR-223-3p as a tumor marker, but it may be worth testing because of its role in tumorigenesis [91,96].

#### 2.7.8. miR-449

miR-449a and miR-449b are highly up-regulated by the transcription factor E2F [97]. E2F has functions in the regulation of the cell cycle and in the induction of apoptosis. Pro-apoptotic genes are usually induced by DNA damage, similar to p53 stimulus [98]. miR-449a can reduce the expression of cell cycle protein CDK6, thereby counteracting cell cycle progression [99]. High miR-449a expression was found in healthy testicular tissue and also in the lung and trachea, while in testicular cancer, expression was low or absent. This may be due to a mutation of retinoblastoma protein (pRB) that binds to E2F. In further consequence, silencing of miR-449a may occur in tumor cells, allowing further progression [97,100]. Nevertheless, a silenced miRNA in tumor tissue won´t have any utility as a circulating tumor marker.

#### 2.7.9. miR-383

miR-383 affects apoptosis, cell cycle regulation and proliferation of TGCTs. While decreased expression has been found in infertile men, expression is elevated in embryonic carcinomas. miR-383 interacts directly with mRNA of interferon regulatory factor-1 (IRF1), whereby the expression decreases. In further consequence IRF1 interacts with cyclin D1, CDK2 and p21, so that at high miR-383 levels the expression of these cell cycle proteins decreases. As previously described with miR-449, miR-383 can also regulate the pRB protein in embryonic carcinomas via interacting with IRF1 [101].

miR-383 overexpression in embryonic carcinomas leads to the inhibition of the DNA damage marker γ-H2A.X, which increases the tumor’s sensitivity to cisplatin. This may be a potential target for the treatment of embryonic carcinomas [102].

#### 2.7.10. miR-514a-3p

miR-514a-3p is part of the miR-506~514 cluster and induces apoptosis by direct interaction with paternally expressed genes 3 (PEG3) and further activation of NF-κB. Downregulation of miR-514a-3p was observed in seminomas and embryonal carcinomas. miR-514a-3p acts proapoptotically by activating p53 via PEG3 and Siah1 and inhibiting the Wnt signaling cascade [103,104]. Overexpression of miR-514a-3p and increased inhibition of PEG3 slow down TGCT’s apoptotic mechanisms, thereby playing an important role in tumor development [105].

#### 2.7.11. miR-199a-3p/214

Alterations in tumor metabolism (aerobic glycolysis) are hallmarks of cancer. The increased conversion of pyruvate to lactate by tumor cells is characterized by a poor prognosis. miR-199a-3p increases lactate production by downregulating metabolic genes. In TGCTs miR-199a-3p is therefore commonly found at high expression levels [106]. Another direct target of miR-199a is V-maf musculoaponeurotic fibrosarcoma oncogene homolog B (MAFB)). The low expression of this protein may explain the antiproliferative properties of miR-199a. Interestingly, expression of miR-199a is high in TGCT, whereas it is decreased in glioblastomas compared to healthy tissue [107].

Furthermore, miR-199a-3p was shown to be negatively correlated with the expression of DNA (cytosine-5)-methyltransferase 3A (DNMT3A), thereby affecting methylation patterns in TGCTs [108]. miR-199a-3p is a potential target for treatment because of its tumor suppressive properties, however, there are no studies on its potential role as a biomarker [106]. 

**Figure 1:**
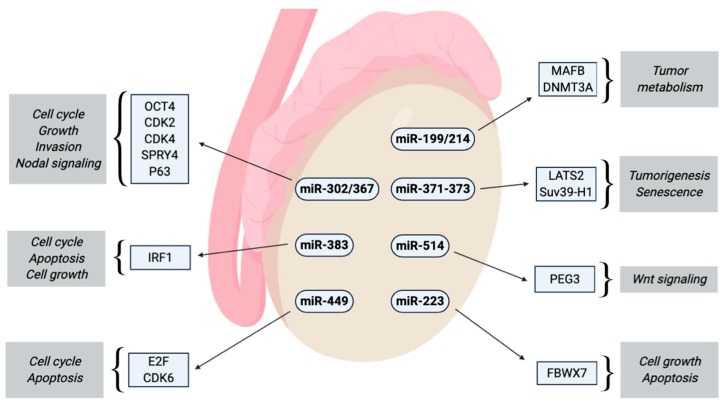
Summary of the effects of miRNAs with altered expression in TGCTs.

miR-214 is co-transcribed together with miR-199a-2 and accordingly down-regulated in TGCTs. Both miRNAs inhibit the expression of DNMT1, whereas only miR-214 can activate TP53 [109]. In overview about all miRNAs and their potential interactions with driving and tumor suppressive factors is depicted in Figure 1.

## 3. Conclusions

This work has highlighted the limitations of currently used tumor markers, namely AFP, β-hCG, and LDH. Relatively low sensitivity and specificity and the influence of other tumors or diseases are the main reasons why conventional serum markers are far below perfect. In addition, epigenetic changes such as DNA methylation, histone modifications, or miRNAs carry great potential that can revolutionize the diagnosis of testicular tumors. miR-371a-3p has been analyzed in many studies, including multicenter studies, and harbor the greatest potential to get established as a marker that can be used in monitoring disease and detection of disease recurrence. miR-302 and miR-367 are also highly sensitive, but have more limitations and may serve as an additional marker to miR-371a-3p.

Many other miRNAs (miR-223-3p, miR-449, miR-383, miR-514a-3p, miR-199a-3p, miR-214) change their expression pattern during the transformation to malignant testicular tumors and may be considered as potential tumor markers or therapeutics. The further investigation of these miRNAs in the future is certainly warranted. The established miRNA, miR-371a-3p, seems to be the first one ready for prime time in assessing presence of metastatic disease and monitoring treatment success in cisplatin-treated TGCTs patients. In other disease settings, especially teratomas, and for further miRNAs, additional prospective large studies are needed to confirm their potential as tumor markers and to better understand the limitations of these novel serum markers.
